# Targeting Plk1 with siRNNs in primary cells from pediatric B-cell acute lymphoblastic leukemia patients

**DOI:** 10.1038/s41598-020-59653-5

**Published:** 2020-02-14

**Authors:** Oksana Goroshchuk, Linda Vidarsdottir, Ann-Charlotte Björklund, Alexander S. Hamil, Iryna Kolosenko, Steven F. Dowdy, Caroline Palm-Apergi

**Affiliations:** 1 Department of Laboratory Medicine, Clinical Research Center, Karolinska Institutet, Stockholm, Sweden; 2 Department of Cellular & Molecular Medicine, UCSD School of Medicine, La Jolla, California, USA

**Keywords:** Acute lymphocytic leukaemia, Drug delivery, B cells

## Abstract

B-cell acute lymphoblastic leukemia (B-ALL) accounts for nearly one fifth of all childhood cancers and current challenges in B-ALL treatment include resistance, relapse and late-onset side effects due to the chemotherapy. To overcome these hurdles, novel therapies need to be investigated. One promising target is Polo-like kinase 1 (Plk1), a key regulator of the cell cycle. In this study, the Plk family expression is investigated in primary peripheral blood and bone marrow mononuclear cells from ten pediatric B-ALL patients. For the first time, short interfering RiboNucleic Neutrals (siRNNs) that enter cells without a transfection reagent are used to target Plk1 mRNA in primary cells from pediatric B-ALL patients. Our results show that the expression of Plk1 and Plk4 is significantly higher in pediatric B-ALL patients compared to healthy donors. Moreover, treatment of primary peripheral blood and bone marrow mononuclear cells from pediatric B-ALL patients, cultured *ex vivo*, with Plk1-targeting siRNNs results in cleavage of Plk1 mRNA. Importantly, the Plk1 knockdown is specific and does not affect other Plk members in contrast to many small molecule Plk1 inhibitors. Thus, Plk1 is a potential therapeutic target in pediatric B-ALL and selective targeting of Plk1 can be achieved by the use of siRNNs.

## Introduction

B-cell acute lymphoblastic leukemia (B-ALL) is one of the most common hematological malignancies among children and corresponds to approximately 85% of all pediatric ALL cases^1^. Although more than 80% of pediatric B-ALL patients reach complete remission after the primary induction therapy, there are still many patients who suffer from resistance and relapse. An aggressive form of the disease causes relapse in 10–15% of treated children and 2% are refractory to the induction therapy^2^. The event-free survival of patients with first relapse remains poor and many pediatric patients suffer from therapy-related side effects several years after treatment. Thus, novel therapies need to be investigated to improve outcome and to reduce toxic side effects in patients with pediatric B-ALL.

The Polo-like kinase family (Plk) consists of five serine/threonine kinases. Four members of the family, Plk1–4, are directly involved in cell cycle regulation in both normal and tumor cells, whereas the fifth member, Plk5, has a truncated, inactive kinase domain and is primarily expressed in brain tissue^3^. Several structural features are shared within the Plk family including a kinase domain with an ATP-binding pocket and a polo-box domain^4^. Inhibitors targeting either of the domains have been investigated in many preclinical studies^5^. The most well-studied member, Polo-like kinase 1 (Plk1), has a number of distinct roles in cell cycle regulation such as G2/M transition, mitotic spindle assembly and cytokinesis that all promote cell cycle progression^6–8^. Due to these functions, Plk1 expression is high in actively dividing tissues and often overexpressed both in solid tumor and hematologic malignancies^9–12^. Similarly to Plk1, Plk4 was recently found to be aberrantly expressed and involved in tumor progression^13–15^. Both Plk1 and Plk4 have been investigated as potential targets in anticancer therapies as inhibition of their activity induces cell death in cancer cells. Small molecule inhibitors targeting Plk1 have been investigated in many preclinical studies and a few are currently in clinical trials^5^. However, specificity may be an issue as fatal side effects have been reported for at least one of them^16^. Therefore, we set out to investigate the potential of a more specific inhibition of Plk1 by short interfering RiboNucleic Neutrals (siRNNs).

RNA-interference (RNAi) is a highly specific post-transcriptional gene silencing mechanism induced by short interfering RNAs (siRNAs)^17,18^. In the cytoplasm, the siRNA molecule is loaded into the RNA-induced silencing complex (RISC), followed by Argonaute 2-mediated cleavage of the passenger (sense) strand and subsequent target mRNA cleavage^19^. The process is highly efficient since one siRNA molecule can induce cleavage of several mRNAs. In our previous T-ALL study^9^, we found that RNAi prodrugs targeting Plk1 could enter primary cells from T-ALL patients and induce knockdown of Plk1 mRNA followed by cell cycle arrest and apoptosis. RNAi prodrugs are modified siRNA molecules that enter cells without a transfection reagent and were named siRNNs as they are covalently coupled to S-acyl-2-thioethyl (SATE) phosphotriester groups that neutralize the phosphodiester backbone^20^. Inside the cell, cytoplasmic thioesterases cleave the thioester bond within the SATE phosphotriester group. The cleavage results in a spontaneous two-step rearrangement of siRNN into siRNA, followed by RISC loading and RNAi. Consequently, treatment with Plk1-targeting siRNNs should result in a reduced Plk1 protein expression followed by cell cycle arrest and apoptosis. Herein, we investigate the Plk family expression and if Plk1-targeting siRNNs are able to induce specific Plk1 mRNA knockdown in primary cells from B-ALL patients.

## Results

### Targeting Plk1 in pediatric B-ALL cell lines

**Figure 1:**
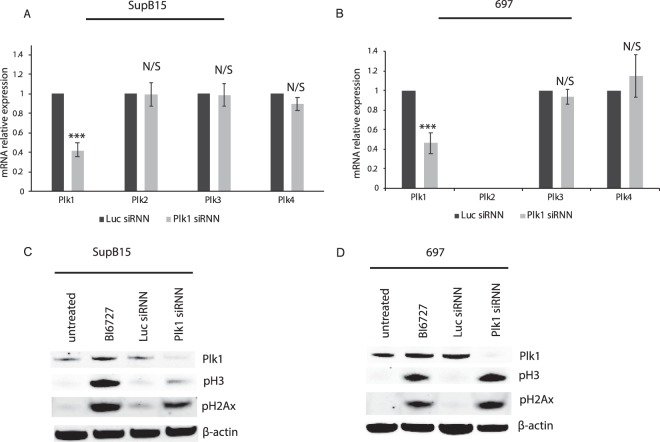
Targeting Plk1 in pediatric B-ALL cell lines. Quantitative RT-PCR analysis of Plk1–4 mRNA expression after Plk1 siRNN treatment (300 nM, 48 h) relative to Luc siRNN treated cells in (**A**) SupB15 and (**B**) 697 cell lines (n = 3/4). ß-actin was used as an internal control. The graphs represent the mean ± standard error of mean (SEM) of at least three independent experiments (***p < 0.001). Western blot analysis of (**C**) SupB15 and (**D**) 697 cells 48 h after treatment with 300 nM Luc/Plk1 siRNN or 25 nM BI6727. Each immunoblot is a representative of at least three independent experiments. Phospho-Histone H3 (pH3) was used as a marker of G2-arrest and phospho-gamma-H2AX indicated double-stranded DNA breaks. ß-actin was used as a loading control. Full-length blots and quantification of blots can be found in Supplementary Figs. 1 and 2, respectively.

First, we investigated if siRNNs targeting Plk1 were able to enter B-ALL cell lines and induce specific mRNA knockdown followed by cell cycle arrest and apoptosis. Treatment with Plk1-targeting siRNNs in two pediatric B-ALL cell lines, SupB15 and 697, induced a ~60% statistically significant (p < 0.001) knockdown of Plk1 mRNA (Fig. 1A,B). To determine the specificity of the Plk1 siRNNs, the expression of Plk2–4 was analyzed after Plk1 siRNN treatment. The mRNA levels of Plk2–4 in SupB15 cells were not affected by the Plk1 siRNN treatment. In 697 cells, Plk2 mRNA could not be detected either with or without treatment, whereas the Plk3 and Plk4 mRNA expression varied insignificantly.

Further, we assessed the protein expression of Plk1, G2 arrest marker phospho-Histone H3 (pH3) and DNA-damage marker phospho-gamma-Histone2AX (pH2AX) after siRNN treatment (Fig. 1C,D) by western blot (full length blots and quantification of blots are presented in Supplementary Figs. 1 and 2, respectively). Plk1 protein was reduced in both cell lines after Plk1-targeting siRNN treatment. The expression of pH3 and pH2AX in Plk1 siRNN treated cells increased, indicating G2/M-phase arrest and DNA-double-strand breaks, respectively. Similarly, B-ALL cells treated with a small molecule Plk1 inhibitor, volasertib (BI6727), showed an induction of pH3 and pH2AX, however, together with a slight accumulation of the Plk1 protein. Moreover, flow cytometry analysis of annexin V and propidium iodide stained cells demonstrated that Plk1-targeting siRNNs induce apoptosis and G2/M-phase arrest in pediatric B-ALL cell lines (Supplementary Figs. 3 and 4, respectively). The effect of four additional siRNA sequences targeting Plk1 were analyzed by flow cytometry and western blot to ascertain that the Plk1 knockdown was not sequence specific or due to an off-target effect and that each sequence induced Plk1 knockdown followed by cell cycle arrest and apoptosis (Supplementary Figs. 5 and 6, respectively).

### Plk1-Plk4 expression in pediatric B-ALL patients

**Figure 2:**
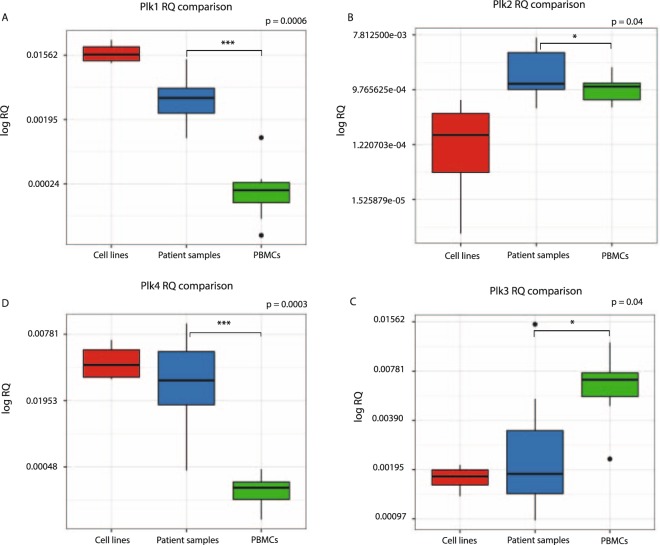
Plk1-Plk4 expression in pediatric B-ALL patients. The boxplot displays median relative mRNA expression of (**A**) Plk1, (**B**) Plk 2, (**C**) Plk3 and (**D**) Plk4 in B-ALL cell lines (red), patient samples (blue) and PBMCs from healthy blood donors (green). The obtained values were plotted as log2 of relative quantification (RQ) values using ggplot. Plk1 and Plk4 expression in primary patient cells was significantly higher (***p = 0.0006/0.0003 respectively) compared to PBMCs. Upper box line represents 75th, middle -50th (median), and lower - 25th percentile (*p < 0.05). Bold black dots represent outliers. GAPDH was used as an internal control.

Next, the mRNA expression of the Plk family members known to be involved in the cell cycle, Plk1–4, was assessed in primary cells from peripheral blood and bone marrow mononuclear cells collected from ten pediatric B-ALL patients within the age of 1–18 years (Table 1). The expression of Plk1–4 was also assessed in six B-ALL cell lines and peripheral blood mononuclear cells (PBMCs) from twelve healthy blood donors (Fig. 2, Supplementary Fig. 7). As expected, B-ALL cell lines had the highest expression of Plk1 among the three groups. B-ALL patients had significantly higher Plk1 expression compared to PBMCs from healthy donors (p = 0.0006, the median in patients was up to 20-fold higher than the healthy control group). The expression of Plk2 and Plk3 was significantly higher and lower between the patients and healthy donors, respectively, when compared as a group (p = 0.04). Interestingly, two B-ALL cell lines, Nalm6 and 697, did not express Plk2 (Supplementary Fig. 7B). Although the Plk4 expression in B-ALL cell lines and patients varied, the Plk4 expression in patients was significantly higher compared to healthy donors (p = 0.0003, up to 8-fold median difference). Overall, the Plk1 and Plk4 expression was clearly higher in tumor cells than in PBMCs from healthy donors compared to the other members of the Plk family.

**Table 1:** Characteristics of pediatric B-ALL patients included in the study.

Patients	Source	Age at diagnosis	Tumor cytogenetics	Years between sampling and experiment	Plk1 mRNA knockdown
1	BM	2	normal	27	54%
2	BM	3	t (12;21)	11	71%
3	BM	1	other	26	30%
4	BM	9	HeH 57, XY	14	N/A
5	BM	11	43, X, −9, −13, −X, +marker 13	4	25%
6	BM	5	HeH	17	N/A
7	BM	18	HeH	15	N/A
8	BM	14	N/A	4	N/A
9	PB	16	normal	27	76%
10*	BM	5	N/A	28	95%

Patients were numbered according to the Plk1 mRNA expression, where patient 1 had the highest Plk1 expression relative to PBMC1 (Supplementary Fig. 7). The last column indicates the level of Plk1 mRNA knockdown after Plk1 siRNN treatment. BM, bone marrow; HeH, high hyperdiploidy; PB, peripheral blood; *SMN, second malignant neoplasm.

Further, we used a publicly available database to analyze the expression of Plk1-Plk4 mRNA in peripheral blood cells from pediatric B-ALL patients in comparison to sorted CD19+ B-cells from healthy donors as described in Materials and Methods (Supplementary Fig. 8). The analysis showed that the expression pattern of Plks between the B-ALL cells and normal B-cells followed the same trend that we observed using our samples in regard to Plk1 (Supplementary Fig. 8A) and Plk4 (Supplementary Fig. 8D) mRNA expression, as they were significantly higher in B-ALL samples compared to sorted B-cells. However, the expression of Plk2 (Supplementary Fig. 8B) and Plk3 (Supplementary Fig. 8C) was lower and higher, respectively, in the patient group compared to the sorted B-cells from healthy donors. In summary, our patient samples and the database expression data correlated in regard to Plk1 and Plk4, as the expression was higher in B-ALL patients than in both PBMCs and in CD19+ sorted B-cells from healthy donors but differed in regard to Plk2 and Plk3 as an opposite expression pattern was found in our patients compared to the database set.

### Targeting Plk1 in primary cells from pediatric B-ALL patients

**Figure 3:**
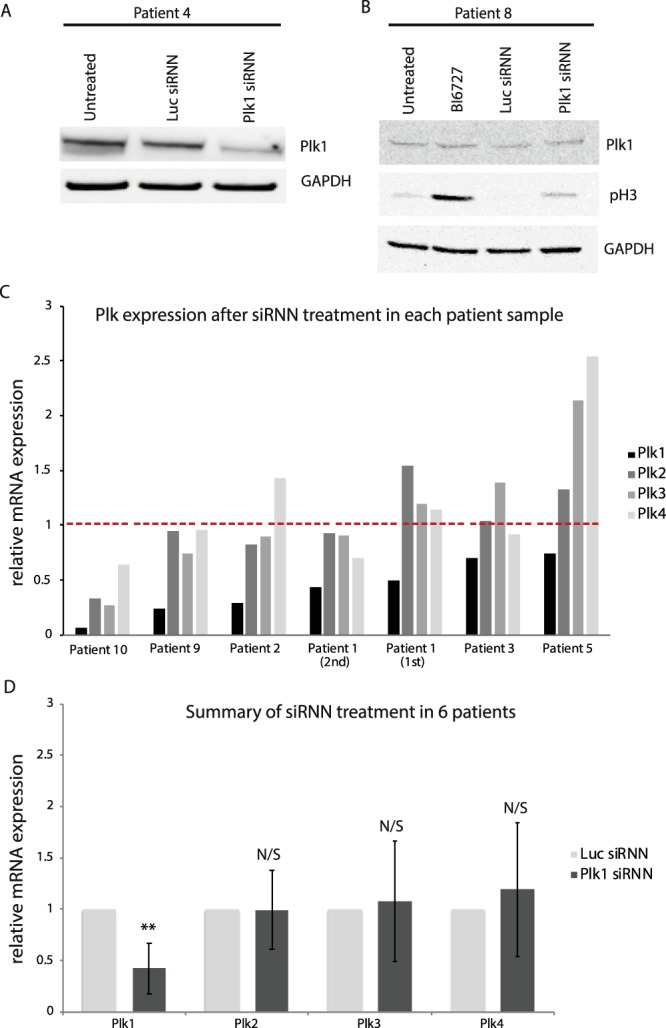
Targeting Plk1 in primary cells from pediatric B-ALL patients. Western blot analysis of Plk1 protein levels in (**A**) Patient 4, 48 h after treatment with Plk1/Luc siRNNs and in (**B**) Patient 8, 24 h after treatment with Plk1/Luc siRNNs or BI6727. The immunoblots represent one independent experiment due to limited number of patient material. In (**A**) Plk1 was detected using Western Lightning Plus-ECL and captured using Kodak M35 X-omat processor whereas GAPDH was developed using Odyssey Infrared Imager. Full-length blots and quantification of blots can be found in Supplementary Figs. 9 and 10, respectively. (**C**) Plk1–4 mRNA expression in primary cells from six B-ALL patients after siRNN-mediated Plk1 knockdown relative to Luc siRNN treatment (red dotted line) within the same patient. The siRNN treatment of primary cells from Patient 1 was performed two times with the interval of 4 days. GAPDH was used as an internal control. (**D**) Combined Plk1–4 mRNA expression in primary cells from six B-ALL patients after siRNN-mediated Plk1 knockdown relative to Luc siRNN treatment. Plk1-targeting siRNNs induced an overall statistically significant Plk1 mRNA knockdown in primary cells from six patients (Supplementary Fig. 11). The expression of Plk2–4 varied insignificantly. Error bars represent mean ± standard deviation (SD) (**p < 0.005).

In order to analyze the effect on Plk1 mRNA and protein knockdown after siRNN treatment, primary patient cells have to be cultured *ex vivo* for at least 24 h. However, there are not many published protocols on how to culture primary cells from B-ALL patients. Therefore, we developed a protocol based on complete medium supplemented with CD40 and IL-2/4/7. Due to the low number of cells (10–20 million) in each patient sample and varying viability of the cells, the effect on proteins after siRNN treatment was analyzed by western blot in three patient samples. A reduction in Plk1 protein 48 h after siRNN treatment could be verified by western blot in Patient 4 (Fig. 3A) (full length blots are presented in Supplementary Fig. 9A,B and quantification of the blots in Supplementary Fig. 10A). In a second patient (Patient 8), treatment with small molecule inhibitor volasertib, resulted in an increase of G2 arrest marker pH3, 24 h after treatment (Fig. 3B) (full length blots are presented in Supplementary Fig. 9C,D). A weak band indicating G2 arrest could be detected in the Plk1 siRNN treated sample and quantification of the blot indicated a decrease of Plk1 that could result in the increase in pH3 (Supplementary Fig. 10B,C). In a third patient (Patient 9), western blot analysis indicated that cell cycle arrest and apoptosis were induced after 24 h as pH3 and cleaved PARP were detected, however, Plk1 knockdown could not be verified on the protein level (data not shown) but only on the mRNA level (Fig. 3C).

We were able to perform qRT-PCR analysis of Plk1–4 after Plk1 or Luc siRNN treatment in primary cells from six pediatric B-ALL patients (Fig. 3C). Treatment with Plk1-targeting siRNNs in Patient 9 (where an increase of G2 arrest and DNA double-strand breaks was detected) induced ~80% knockdown of Plk1 mRNA compared to the negative siRNN control sequence, targeting Luc. An additional five patient samples (Patient 1–3, 5 and 10) were treated with Plk1-targeting siRNNs and analyzed for Plk1–4 mRNA expression with one patient being analyzed in biological duplicates (Patient 1). In total, Plk1-targeting siRNNs induced a Plk1 knockdown greater than 50% in four patient samples, around 30% in two patients and a similar knockdown of 50% in the two independently performed experiments on the sample from Patient 1. Overall, Plk1 siRNN treatment of primary cells led to a statistically significant knockdown of Plk1 mRNA (p < 0.005) compared to the control when combining the six patient samples (Fig. 3D, Supplementary Fig. 11). Importantly, the expression of Plk2–4 was not significantly affected by the Plk1 siRNN treatment.

**Figure 4:**
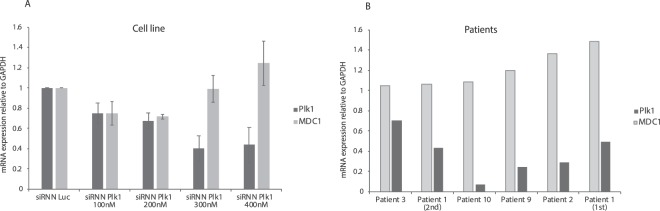
MDC1 analysis in primary cells from pediatric B-ALL patients and cell lines after siRNN treatment. MDC1 mRNA expression in pediatric B-ALL. (**A**) Cell line 697 treated with Plk1 or Luc siRNN at 100–400 nM and analyzed by qRT-PCR for Plk1 and MDC1 expression 24 h after treatment. The graphs represent the mean ± standard error of mean (SEM) of at least three independent experiments (n = 3). GAPDH was used as internal control. (**B**) Five patient samples treated with Luc or Plk1 siRNN at a concentration of 200 nM and analyzed by qRT-PCR for Plk1 and MDC1 expression 24 h after treatment. A significant increase in MDC1 after Plk1 mRNA knockdown was detected when the data from the patient samples were combined (Supplementary Fig. 11). GAPDH was used as internal control. Error bars represent mean ± SEM.

To assess if double-stranded DNA breaks were induced in the patient samples after siRNN-mediated Plk1 mRNA knockdown, in accordance with the western blot data on the cell lines, we analyzed the mediator of DNA damage checkpoint 1 (MDC1) by qRT-PCR in the siRNN-treated patient samples. MDC1 is recruited after the phosphorylation of H2AX (pH2AX) by ataxia telangiectasia mutated (ATM) kinase during a double-stranded DNA break and is therefore, similarly to pH2AX, an indicator of DNA damage. When MDC1 was analyzed in B-ALL cell lines after Plk1 siRNN treatment (Fig. 4A), the mRNA analysis indicated a trend towards a stepwise increase of MDC1 as the Plk1 mRNA decreased. Moreover, a significant increase in MDC1 mRNA (p < 0.01) was detected in the patient samples after Plk1 siRNN treatment when the data from the patient samples were combined (Fig. 4B, Supplementary Fig. 11).

## Discussion

In the present study we investigated, for the first time, if novel siRNNs could enter primary peripheral blood and bone marrow mononuclear cells from pediatric B-ALL patients and induce knockdown of Plk1 mRNA, a key player in the cell cycle and a promising target in cancer therapy. Current treatment regimens of pediatric B-ALL consist of an intensive chemotherapy treatment including vincristine, corticosteroids and anthracyclines followed by  allogeneic stem cell transplantation for eligible candidates^2,21^. Although the long-term survival of pediatric B-ALL patients has increased significantly over the past decades, there are still patients who are resistant to the existing therapy or relapse after the primary treatment. Moreover, several cycles of intensive treatment induce both acute and late-onset side effects, resulting in a decreased quality of life^22^. Therefore, novel targets and tumor specific therapies need to be investigated in pediatric B-ALL.

Out of the five members of the Plk family, Plk1 and Plk4 are considered as promising targets in cancer therapies due to their important functions as kinases during the cell cycle of tumor cells^5,23^. A plethora of preclinical studies have been performed on potential small molecule inhibitors that target either the N-terminal kinase domain or the C-terminal Polo-box domain (PBD), specific to the Plk family. However, the IC_50_ values of the inhibitors targeting the PBD are usually in the micromolar range and up to date there has not been any clinical trials of PBD inhibitors in acute leukemia. In contrast, most of the small molecule inhibitors targeting the ATP-pocket of the kinase domain, have IC_50_ values in the low nanomolar range and several ATP-competitive inhibitors have entered clinical trials^5^.

The most successful Plk1 inhibitor, volasertib (BI6727), reached phase III clinical trials for adult acute myeloid leukemia patients ineligible for intensive remission induction therapy^24^. The drug was applied in a combination with cytarabine. However, the combination treatment led to fatal side effects in some patients. Possible explanations for the side effects may be low specificity, as the IC_50_ values of volasertib are 0.87, 5, and 56 nM in Plk1, Plk2 and Plk3, respectively^25^. There may also be a cross-reactivity of volasertib with other crucial proteins via the ATP-binding domain^26^. In contrast, preclinical studies on genetically-engineered mice showed that specific Plk1 mRNA knockdown by short hairpin RNA did not result in typical hematological side effects, such as anemia or neutropenia, and did not affect spermatogenesis or cellular proliferation^27^. Moreover, our studies have shown that specific Plk1 knockdown by RNAi prodrugs, result in little toxicity in normal cells^9^. Therefore, a more specific targeting of Plk1 may lead to reduced side effects in future therapies.

RNAi therapeutics has taken advantage of the RNAi mechanism to induce gene silencing by sequence-specific cleavage of target mRNAs. In 2018, the U.S. Food and Drug Administration (FDA) approved a new siRNA-based drug, patisiran (Onpattro), to be prescribed for adult patients with rare hereditary polyneuropathy due to amyloidosis^28^. The FDA designated patisiran to Fast Track, Priority Review, Breakthrough Therapy and Orphan Drug applications. Another siRNA-based drug, inclisiran, completed a phase II trial (ORION-1) in participants with elevated low density lipoprotein cholesterol^29^. Inclisiran is a fully chemically modified siRNA conjugated to the triantennary N-acetylgalactosamine that targets the 3′UTR of proprotein convertase subtilisin kexin type 9 mRNA within hepatocytes. The results from the study showed that inclisiran produced a significant and prolonged reduction in atherogenic lipoproteins. In 2019, the FDA approved another N-acetylgalactosamine-based drug, givosiran (Givlaari) to treat acute hepatic porphyria in adults^30^ that is able to induce a reduction in the mRNA of delta aminolevulinic acid synthase *ALAS1*^31^. Monthly injections of givosiran significantly reduced parameters such as *ALAS1* mRNA, urinary porphobilinogen and urinary aminolevulinic acid, and improved clinical manifestations of the disease. Thus, RNAi therapeutics has become reality. However, currently there are no clinical trials on siRNA-based drugs for leukemia treatment.

In this study, we investigated the mRNA expression of Plk1–4 in primary cells from B-ALL patients and if treatment with Plk1-targeting siRNNs resulted in a specific Plk1 knockdown without affecting the other Plk members. As we could not access primary bone marrow and peripheral blood mononuclear cells from healthy children, we used PBMCs from healthy, adult blood donors as a “normal” control. In accordance with our previous T-ALL study^9^, the expression of Plk1 was significantly higher in primary peripheral blood and bone marrow mononuclear cells originating from pediatric B-ALL patients, than in PBMCs from healthy donors. Similarly, and in agreement with previous studies in colorectal cancer, the Plk4 expression was also significantly higher in B-ALL patients compared to normal cells^32^. Furthermore, these findings were supported when the expression of Plk1 and Plk4 was analyzed in publicly available databases, as the expression of Plk1 and Plk4 was found to be significantly higher in B-ALL patients compared to sorted normal B-cells from healthy donors.

Since the functions of Plk5 are not connected to the cell cycle and Plk5 is primarily found in brain tissue, we did not evaluate its expression. Plk2 and Plk3, on the other hand, play many roles in the cell cycle and predominantly act as tumor suppressors^5,33–35^. Thus, they should be downregulated in tumors. Still, our data showed that Plk2 mRNA was expressed in primary cells from pediatric B-ALL patients and several fold higher in four patients compared to the other samples. However, in two of the cell lines, Plk2 could not be detected, which is in line with previous studies in B-cell malignancies where Plk2 was found to be transcriptionally silenced^33,34^. Moreover, the expression of Plk3 was lower in patients compared to healthy donors with the exception of one patient. Interestingly, when Plk2 and Plk3 were analyzed in publicly available databases, the expression pattern of Plk2 and Plk3 was found to be inverse in relation to our patient samples. However, there was a high variability of Plk2 and Plk3 in both patient samples and cell lines and thus, more data on Plk2 and Plk3 expression in tumor cells relative to normal cells need to be gathered.

When Plk1-targeting siRNNs were applied to primary blood and bone marrow mononuclear cells from eight pediatric B-ALL patients, cultured *ex vivo*, Plk1 knockdown was detected in seven of the treated patient samples, independently of genetics or blood/bone marrow origin. Volasertib and Plk1 siRNN treatment of the eighth patient sample led to an induction of G2/M arrest marker pH3 and a decrease of the Plk1 protein could be detected by quantification. In addition, due to the low cell number and poor viability of the primary cells after *ex vivo* culturing, knockdown of the Plk1 protein by siRNNs could only be verified in one patient at 48 h (Patient 4). Although cell cycle arrest and apoptosis could not be analyzed in the resulting six patient samples where Plk1 mRNA knockdown was confirmed, an increase in MDC1 was detected in all six patients indicating the presence of double-stranded DNA breaks. Moreover, the cell line data clearly showed that a reduction of Plk1 led to cell cycle arrest, double-stranded DNA breaks and apoptosis as an increase in pH3, pH2AX and MDC1 were detected. Importantly, mRNA analysis after siRNN treatment confirmed that Plk1 knockdown occurred in all six patients, that the overall knockdown was significant and that it was specific to Plk1 with insignificant effects on Plk2–4.

In conclusion, the expression of Plk1 and Plk4 in pediatric B-ALL patients was significantly higher compared to healthy blood donors and Plk1-targeting siRNNs induced a Plk1 specific and significant knockdown in each treated patient sample. Thus, Plk1 is a promising target for future B-ALL therapies and by using RNAi therapeutics a high degree of specificity may be achieved.

## Methods

### Cell lines and reagents

Cell lines were obtained from ATCC, recently authenticated and routinely checked for mycoplasma contamination. Cell lines were cultured in a humidified incubator at 37 °C with 5% CO_2_. Media (RPMI-1640) for cell culturing was supplemented with 10% FBS, 2mM L-glutamine, 100 μg/mL streptomycin and 100 U/mL penicillin (Nordic Biolabs, Stockholm, Sweden). SupB15, Nalm6 and RS4;11 cell lines were cultured in RPMI-1640 with Hepes from Gibco; 697 was cultured in RPMI-1640 from HyClone; SEM was cultured in RPMI-1640 with Hepes and without antibiotics (Nordic Biolabs, Stockholm, Sweden) and U2OS was cultured in high glucose DMEM (Life Technologies). BI6727 (Volasertib) was purchased from Scandinavian Medical Service (Stockholm, Sweden).

### Primary cells from B-ALL patients and healthy blood donors

B-ALL samples were collected in Astrid Lindgren’s Children Hospital (Karolinska University Hospital) at the time of primary diagnosis (confirmed by a pathologist). Informed consent was obtained from the patients or legal guardians. Ethical approval was granted by the regional ethical committee in Stockholm, Sweden. At the time of sampling, mononuclear cells from bone marrow and/or peripheral blood were isolated by centrifugation on a Ficoll/Hypaque gradient and cryo-preserved in liquid nitrogen in accordance with the relevant guidelines and regulations. Primary mononuclear cells originating from peripheral blood or bone marrow from ten patients diagnosed with B-ALL were cultured *ex vivo* in complete RPMI-1640 Gibco with Hepes (Nordic Biolabs, Stockholm, Sweden), 10% FBS, 2 mM L-glutamine, 100 µg/ml streptomycin and 100 U/ml penicillin (all from Nordic Biolabs). Additionally, media was supplemented with recombinant IL-2 (100 U/mL), recombinant IL-4 (100 U/mL), recombinant IL-7 (1 ng/mL) and CD40-ligand (CD40L) (100 µg/mL) (Tebu-bio, Denmark). Peripheral blood mononuclear cells from healthy blood donors were used as a normal control for the mRNA analysis of Plk1–4 expression.

### RNA interference

Double-stranded siRNAs oligonucleotides were ordered from Integrated DNA Technologies (IDT, USA.) and synthesized with the guide sequences:

(5′-ACCUUUUCCUGAAUGAAGAUCUGGA-3′, Plk1_IDT1 siRNA);

(5′-GACGAGUUCUUUACUUCUGGCUATA-3′, Plk1_IDT2 siRNA);

(5′-AAUAUUUCUAUUGAAUUCGGAACTG- 3′, Plk1_IDT3 siRNA).

Double-stranded siRNAs oligonucleotides with the sequences

(5′- CGAGCUGCUUAAUGACGAGUU-3′, Plk1_1) and

(5′-UCUGUCUGAAGCAUCUUCUUT-3′, Plk1_5) were synthesized as described previously^20^.

Lipofectamine 2000 reagent was purchased from Life Technologies.

For transfection with siRNA, U2OS cells were plated in 6-well plates at 150,000 cells/well. 300 µL of corresponding siRNA (100 nM as a final concentration) in serum-free Opti-MEM (Life Technologies) were mixed with 300 µL of transfection mix (1 µL Lipofectamine 2000 per 50 µL Opti-MEM). The mixture was left for 30 min at room temperature. Before transfection, cells were washed with PBS and serum-free Opti-MEM. Cells were treated with for 4 h in standard incubation conditions, then the transfection media was removed and 2 mL of complete DMEM was added. After 48 h cells were washed, trypsinized and harvested for western blot and cell cycle analysis.

siRNNs against Plk1 and Luciferase (Luc) were synthesized as previously reported^20^. For all siRNN experiments sequence Plk1_5 was used. 697 and SupB15 cells were seeded onto 6-well plates at the concentrations of 0.2 × 10^6^ and 0.4 × 10^6^ cells/ml, respectively. The next day, cell media was removed and siRNNs, diluted in Opti-MEM to a concentration of 300 nM in a final volume of 500 µL per 1 × 10^6^ cells, were added to the cells without any transfection reagent. After 3 h of incubation under normal growth conditions regular media was added to reach the initial volume.

Patient samples were thawed and put in complete media supplemented with CD40L and interleukins and seeded onto 6-well plates at the concentration of 0.5 × 10^6^ cells/mL. At the time of treatment, siRNNs were diluted in serum-free Opti-MEM to a concentration of 200 nM in a final volume of 500 µl per 1 × 10^6^ cells and added to patient cells without any transfection reagent. Interleukins and CD40L were added to Opti-MEM in the same concentrations as applied for complete media. Cells were incubated under normal growth conditions for 3 h and were slightly shaken every 30 min. After the incubation, complete media containing same concentrations of CD40L, IL-2, IL-4, and IL-7, was added followed by analysis 24 or 48 h after treatment.

### Western blot and antibodies

Cell pellets were lysed in a modified RIPA buffer (50 mM Tris-HCL pH 7.4, 150 mM NaCl, 1 mM EDTA, 1% NP-40 and 1% Glycerol, all from Sigma Aldrich AB) with DTT, phosphatase inhibitor, phosphoSTOP and protease inhibitor cOmplete (Roche), incubated 20–30 min on ice and centrifuged at 7000 rpm for 10 min to remove cell debris. The protein absorbance was measured using Bradford assay (Bio-Rad Laboratories) on spectrophotometer SpectraMax i3X (Molecular Devices). 10–20 µg of total protein was loaded onto 4–12% or 12% Bis-Tris gels (NuPAGE, Life Technologies). The PVDF membranes were blocked in Odyssey Blocking Buffer (LI-COR Biosciences, Lincoln, Nevada, USA) and incubated with primary antibodies at 4 °C overnight. After 2 h incubation with secondary antibodies in TBS with 0.1% Tween-20 (TBST) and 0.01% sodium dodecyl sulfate (SDS), membranes were developed using Odyssey Infrared Imager (LI-COR Biosciences). Alternatively, membranes were incubated with primary antibodies diluted in blocking agent over night at 4 °C and then for 1 h with secondary antibodies at room temperature (HRP-conjugated anti-rabbit from Cell Signaling Technology, #7074). The proteins were detected using Western Lightning Plus-ECL (PerkinElmer) and captured using Kodak M35 X-omat processor. The following primary antibodies were used; β-actin (#A5441, Sigma-Aldrich), GAPDH (#ab8245, Abcam), Plk1 (#4513), Phospho-Histone H3 (Ser10) (#3377) and Phospho-Histone H2AX (Ser139) (#2577), all from Cell Signaling Technology. Secondary antibodies were purchased from LI-COR IRDye®: Goat anti-mouse 800CW (926-32210), Goat anti-rabbit 800CW (926-32211) (Lincoln, Nevada, USA). Quantification was performed using publicly available software Image J (1.50c4) and normalized against β-actin or GAPDH as a loading control. Calculations were performed in Excel to show the ratio between “net protein” and “net loading control” (background quantified and excluded). Data represents mean values of two to four blots ± standard error of mean (SEM).

### Quantitative real-time reverse transcriptase PCR (qRT-PCR)

RNA was extracted from cells using Qiagen RNeasy Mini Kit according to manufactures instructions. Cells, 0.5–1.0 × 10^6^, were harvested, washed in PBS, centrifuged and lysed in the RLT buffer supplemented with dithiothreitol (DTT), further centrifuged, and lysate was mixed with 70% ethanol. Next, samples were transferred to a spin column and centrifuged. The spin columns were washed with RW1 and RPE buffers. As a final step, we washed lysate with RNase-free water, collected the obtained RNA and quantified the amount of RNA using spectrophotometer NanoDrop 2000 (ThermoScientific).

400 ng RNA was used to generate cDNA using High Capacity cDNA Reverse Transcription Kit (Thermo Fisher Scientific) according to the manufacturer’s instructions. qRT-PCR was performed using TaqMan Gene expression assays (Thermo Fisher Scientific) and the CFX96 C1000™ Real-Time System Touch™ Thermal Cycler (BioRad) at the following conditions: 95 °C for 20 sec, 40 cycles of 95 °C for 1 sec and 60 °C for 20 sec. The following probes were used (all purchased from Thermo Fisher Scientific): Plk1 (Hs00153444_m1), Plk2 (Hs01573405_g1), Plk3 (Hs00177725_m1), Plk4 (Hs00179514_m1) and MDC1 (Hs00206182_m1). The expression was standardized to the internal controls ACTB (Hs99999903_m1) or human GAPDH (Hs03929097_g1). Experiments on the cancer cell lines were performed in biological triplicates. Data analysis was performed in CFX Maestro Software (BioRad). The fold gene expression was calculated using the 2^−ΔΔCT^ method (as described by Applied Biosystems).

### Cell cycle and apoptosis analysis

For cell cycle analysis cells were harvested and fixed in ice-cold 75% ethanol and kept at −20 °C overnight. After washing with PBS, cells were stained with propidium iodide (PI) 10 ng/ml and 0.2 mg/ml Ribonuclease A (all from Sigma-Aldrich) in PBS. Apoptosis analysis by Annexin V/PI staining was performed on live cells according to the manufacturer’s instruction (AnnexinV-Fluos from Roche and PI from Sigma-Aldrich). Briefly, cells were harvested, washed in ice-cold PBS and resuspended in Annexin V/PI staining buffer (10 mM Hepes, 140 mM NaCl, 5 mM CaCl_2_ × 2H_2_O). Further, Annexin V-Fluos and PI were added (both 10 ng/mL in 100 µl PBS per sample). Stained cells were analyzed using a CellStream flow cytometer (Merck) and FlowJo software (Tree Star Inc.)

### Expression analysis in R2 database

R2 Genomics analysis visualization platform (R2: Genomics Analysis and Visualization Platform^36^ was used to compare Plk1-Plk4 expression between peripheral blood of B-ALL patients and CD19+ sorted B-cells from healthy individuals. For B-ALL, we used T ALL (B)-Murphy dataset^37–39^, where we selected ‘peripheral blood’ track (n = 76). For sorted B-cells, we utilized the track ‘b_cont’ (n = 9) that refers to B cells from the healthy control group in the dataset ‘CD4-T and B cells (Lauwerys)’^40^. Statistical analysis was performed using ANOVA test within the R2 platform.

### Statistical analysis

Experiments done on the cell lines were performed at least three times and the figures represent mean ± standard error of mean (SEM). Package “ggplot”^41^ was used to create boxplot with log2 (fold change) of relative quantification values of mRNA expression in different groups of samples. Statistical analysis of the data was performed using Student’s t-test (two-sided) and ANOVA.

## Supplementary information

Supplementary Infomation.

## Data Availability

The datasets generated during and/or analyzed during the current study are available from the corresponding author on reasonable request.
