# Supplementary material for: Targeting Plk1 with siRNNs in primary cells from pediatric B-cell acute lymphoblastic leukemia patients
Source: Sci Rep. 2020 Feb 14;10:2688. doi: 10.1038/s41598-020-59653-5 (PMC7021816; doi:10.1038/s41598-020-59653-5)
Supplement: Supplementary file 1 — Supplementary Infomation. [file 41598_2020_59653_MOESM1_ESM.pdf]

Targeting Plk1 with siRNNs in primary cells from pediatric B-cell acute lymphoblastic leukemia patients

Oksana Goroshchuk[1], Linda Vidarsdottir[1], Ann-Charlotte Björklund[1], Alexander S. Hamil[2], Iryna Kolosenko[1], Steven F. Dowdy[2] and Caroline Palm-Apergi[1]*

[1]Department of Laboratory Medicine, Clinical Research Center, Karolinska Institutet, Sweden.
[2]Department of Cellular & Molecular Medicine, UCSD School of Medicine, La Jolla, California, USA.

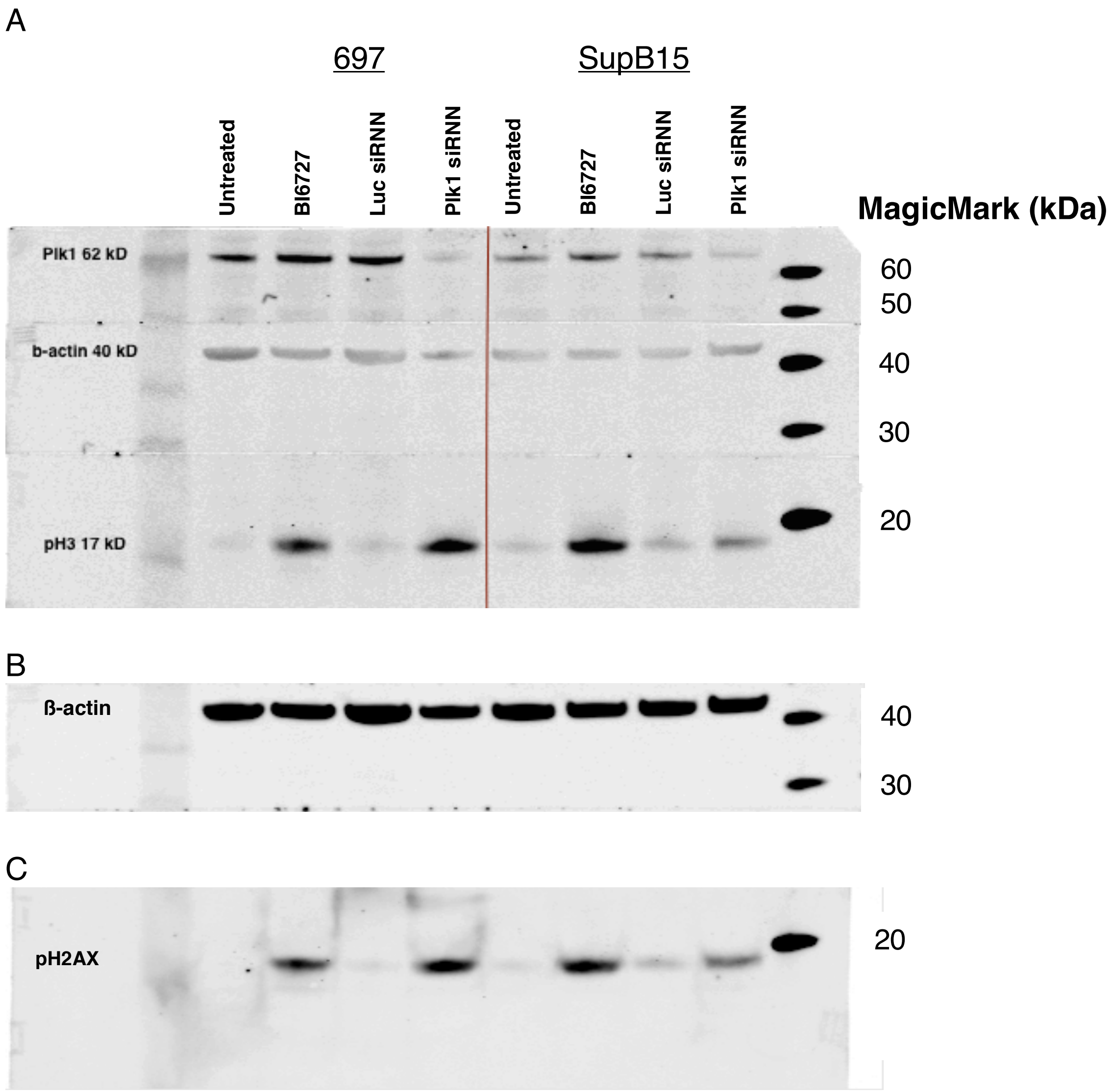

Supplementary Figure 1. Targeting Plk1 in pediatric B-ALL cell lines.

Full-length blots of cropped membranes displayed in Figure 1C and 1D. The blots originate from the same gel and membrane. The ladders of SeeBlue and MagicMark can be detected on the left and right side of blot, respectively. After transfer the membrane was cut into the three parts corresponding to the molecular weights of Plk1, pH3/pH2AX and ß-actin followed by probing for the corresponding antibodies (A). After the first blotting the ß-actin part of the membrane was reprobed with fresh antibody targeting ß-actin (B). The lower part of the membrane was stripped and probed with an antibody targeting pH2AX (C).

A

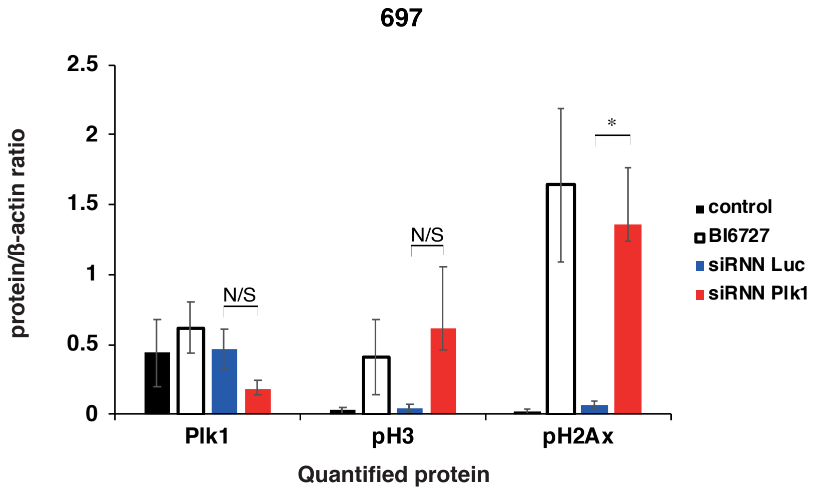
697
protein/ß-actin ratio
2.5
2
1.5
1
0.5
0
N/S
N/S
*
Plk1
pH3
pH2Ax
Quantified protein
control
BI6727
siRNN Luc
siRNN Plk1

B

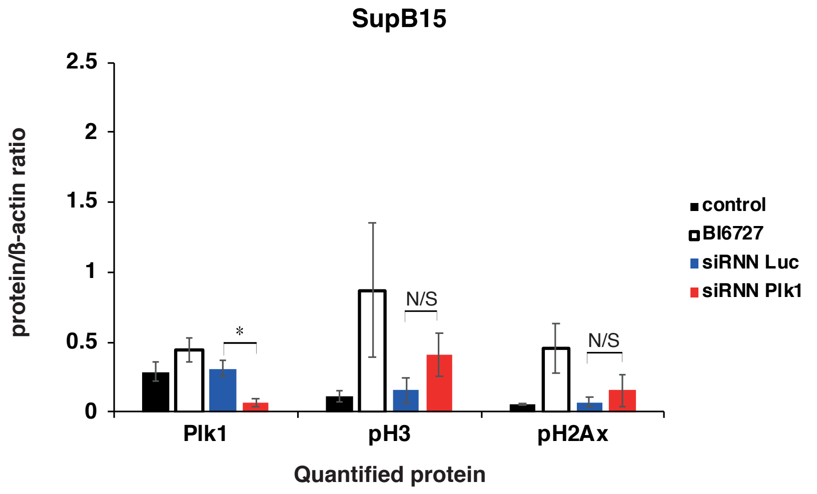
SupB15
protein/ß-actin ratio
2.5
2
1.5
1
0.5
0
*
N/S
N/S
Plk1
pH3
pH2Ax
Quantified protein
control
BI6727
siRNN Luc
siRNN Plk1

C

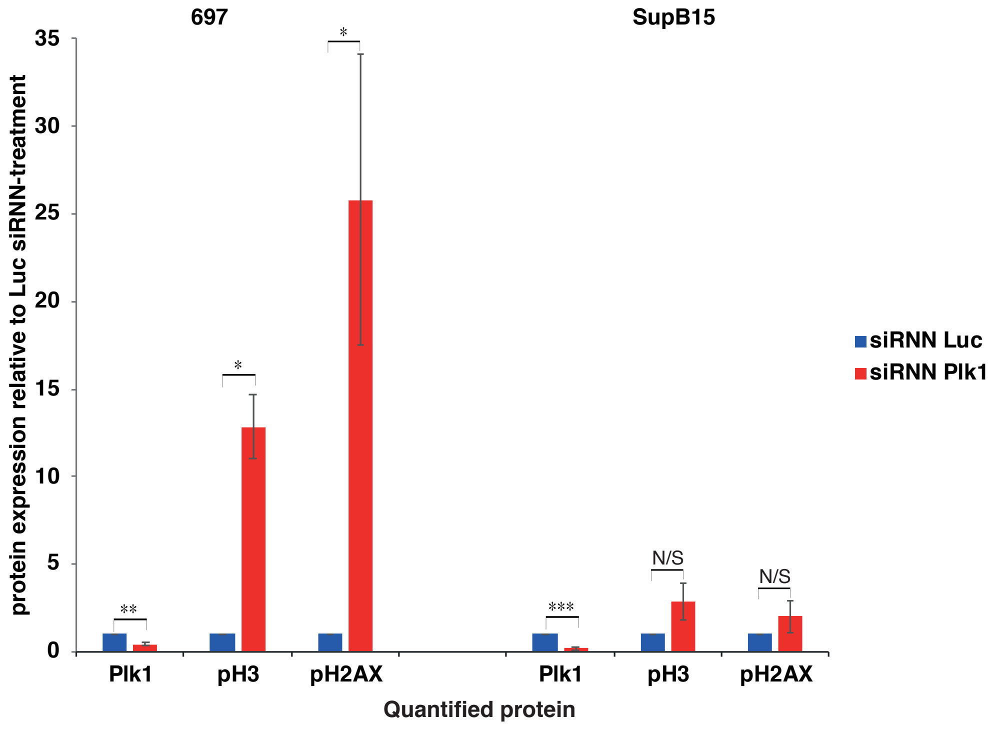
697
SupB15
protein expression relative to Luc siRNN-treatment
35
30
25
20
15
10
5
0
**
*
*
***
N/S
N/S
Plk1
pH3
pH2AX
Plk1
pH3
pH2AX
Quantified protein
siRNN Luc
siRNN Plk1

Supplementary Figure 2. Quantification of western blots performed on B-ALL cell lines. Quantification of four independent western blot experiments analyzed using Image J. Protein expression of Plk1, pH3 and pH2AX was normalized against β-actin. Data represents mean band intensity of quantified proteins before and after treatment with 25 nM BI6727, 300 nM Luc siRNN and 300 nM Plk1 siRNN of at least three independent experiments ± SEM. (A-B) Bar chart A and B are displayed in absolute values and (C) bar chart C represents quantified bands relative to Luc siRNN where Luc siRNN was set to 1. Plk1 siRNN induced a statistically significant Plk1 protein knockdown at 48 h in both cell lines compared to Luc siRNN-treated cells (*p<0.05, **p<0.005, ***p<0.001).

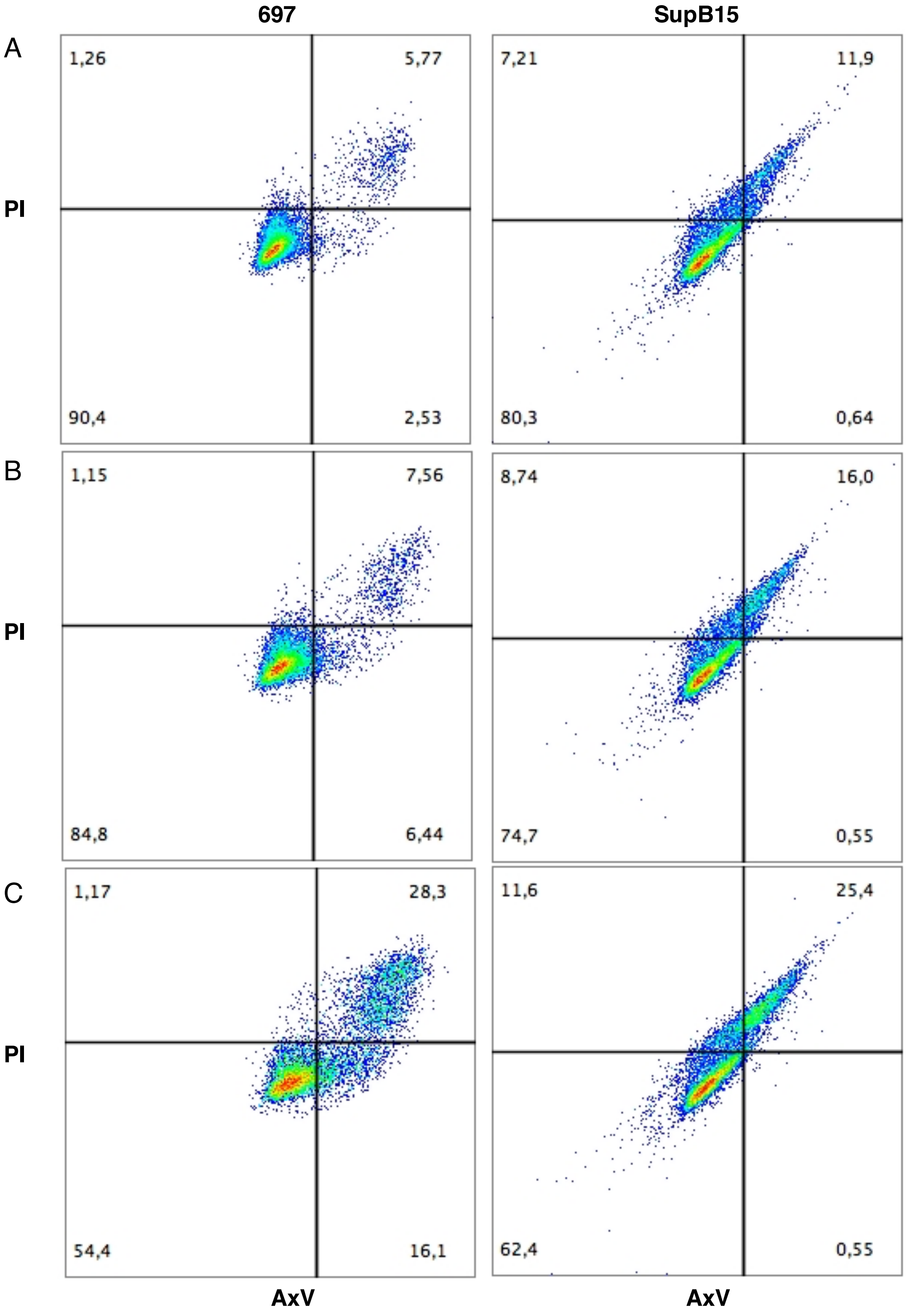
697
SupB15
A
B
C
PI
PI
PI
AxV
AxV
1,26
5,77
90,4
2,53
7,21
11,9
80,3
0,64
1,15
7,56
84,8
6,44
8,74
16,0
74,7
0,55
1,17
28,3
54,4
16,1
11,6
25,4
62,4
0,55

D

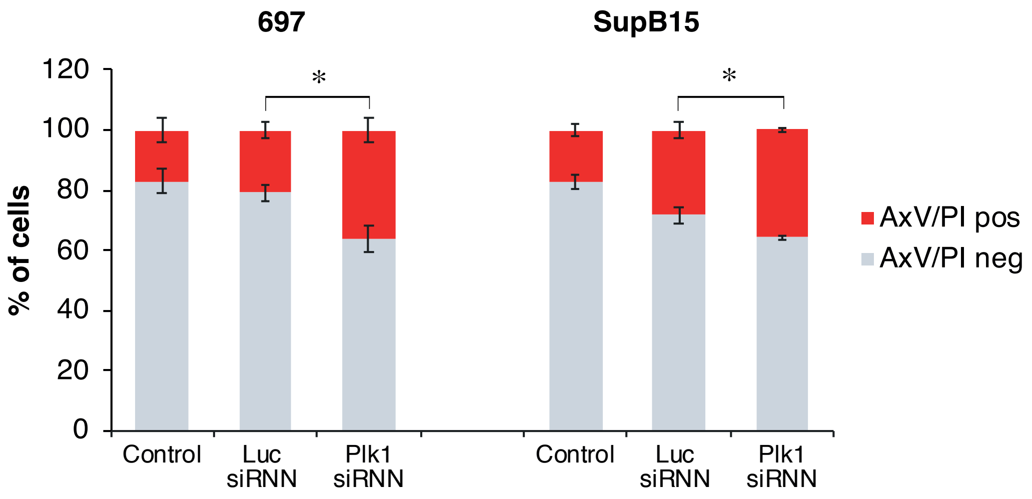

Supplementary Figure 3. AxV/PI staining of siRNN treated pediatric B-ALL cells. Pediatric B-ALL cell line 697 (left column) and SupB15 (right column) were stained with AxV/PI and analyzed by flow cytometry 48 h after treatment with 300 nM siRNNs. Control (A), Luc siRNN (B), Plk1 siRNN (C). Each dot plot is a representative of three independent experiments summarized in (D). Data quantification showed that Plk1 siRNN significantly increased the number of AxV/PI-positive cells (red) in both cell lines (n=3± SEM) (*$p<0.05$).

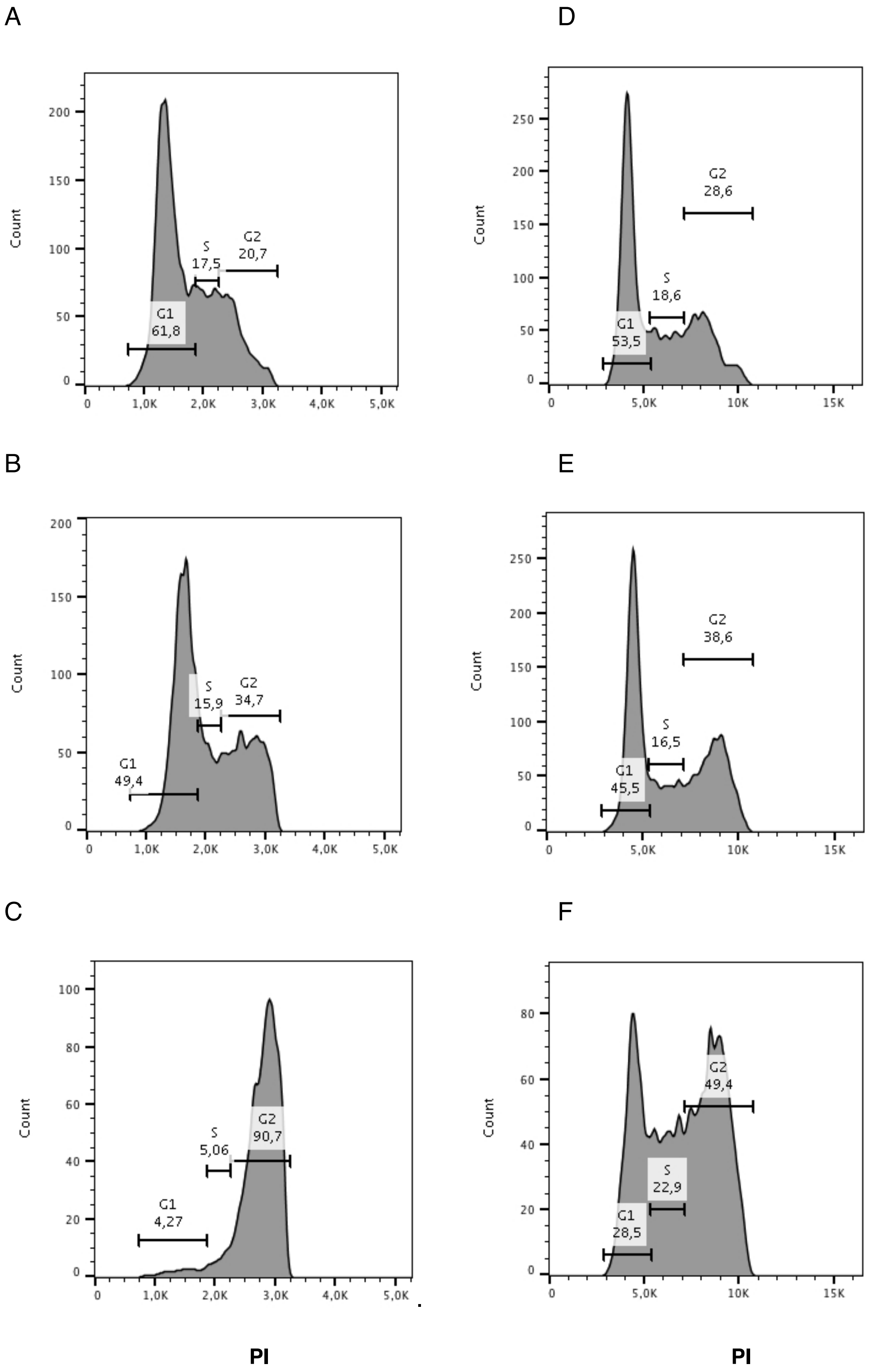
A
B
C
D
E
F
Count
PI
G1 61,8
S 17,5
G2 20,7
G1 49,4
S 15,9
G2 34,7
G1 4,27
S 5,06
G2 90,7
G1 53,5
S 18,6
G2 28,6
G1 45,5
S 16,5
G2 38,6
G1 28,5
S 22,9
G2 49,4
0
1,0K
2,0K
3,0K
4,0K
5,0K
10K
15K

G

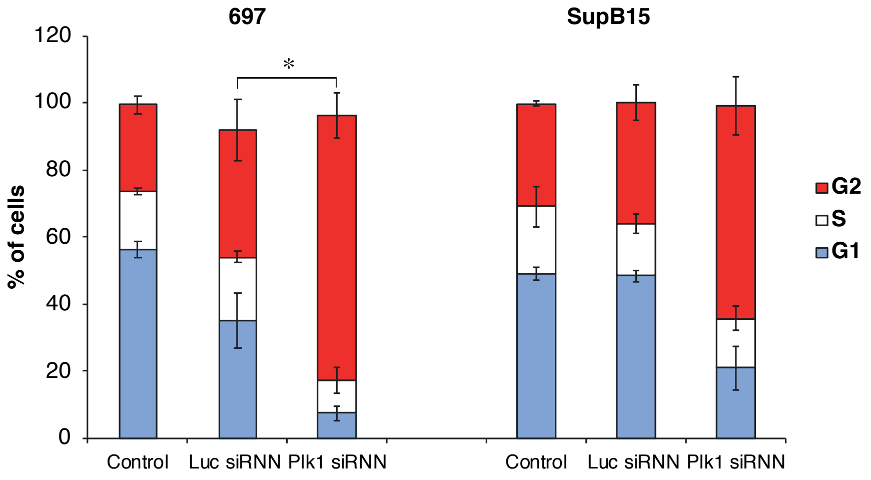

Supplementary Figure 4. PI staining of siRNN treated pediatric B-ALL cells.

Pediatric B-ALL cell lines 697 (A-C) and SupB15 (D-F) were stained with PI and analyzed by flow cytometry 48 h after treatment with 300 nM Plk1 (C, F) or Luc (B, E) siRNNs or control (A, D). Each histogram is a representative of three independent experiments (n=3) ± SEM summarized in (G). Plk1 siRNN significantly (*$p<0.05$) increased the number of G2-arrested cells (red) in both cell lines compared to Luc siRNN-treated cells.

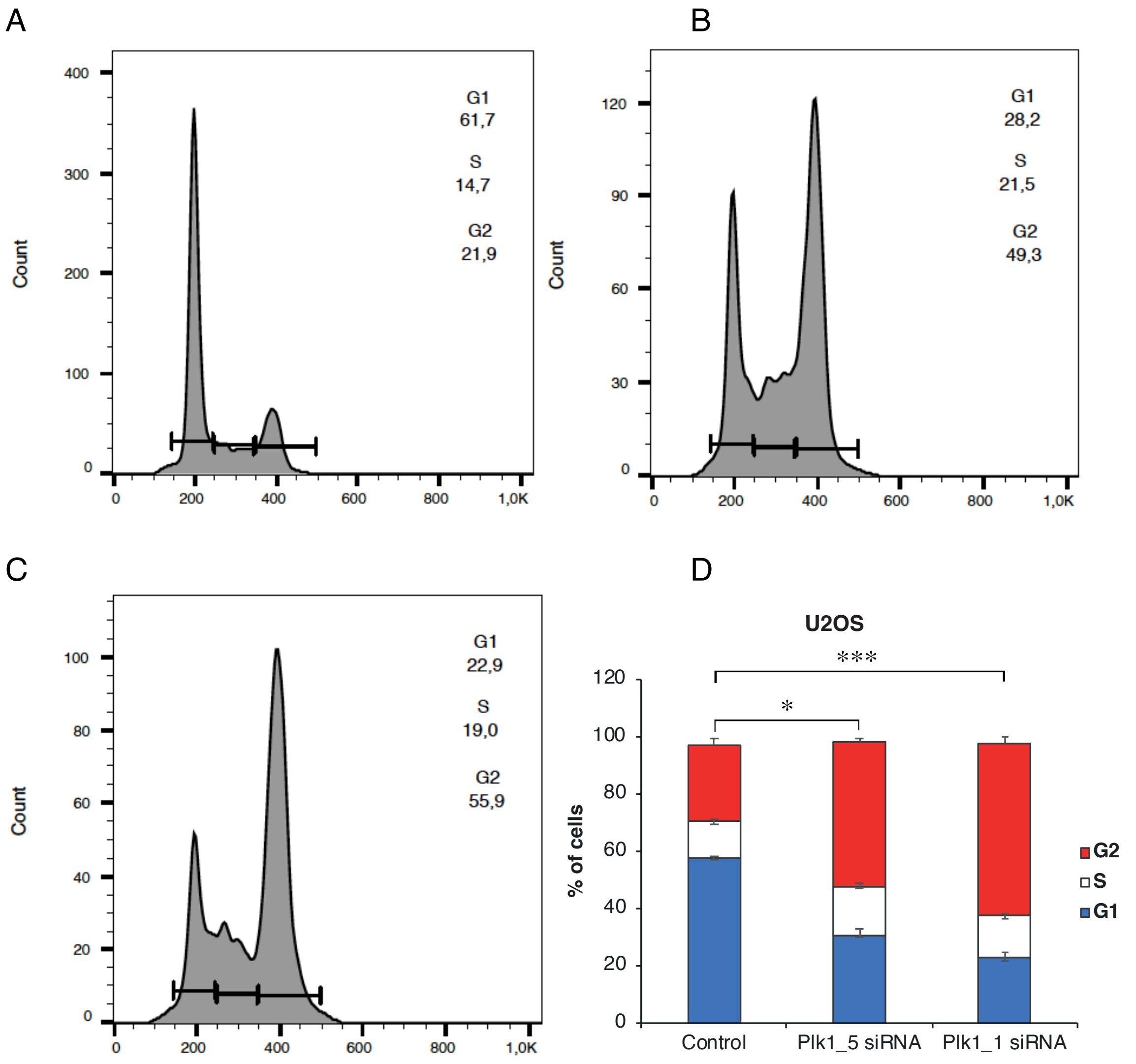

Supplementary Figure 5. Cell cycle analysis of siRNA sequences Plk1_5 and Plk1_1. Pediatric osteosarcoma cell line U2OS treated with two different sequences targeting Plk1, Plk1_5 (B) and Plk1_1 (C) or control (A) followed by PI staining and flow cytometry analysis 48 h after treatment with 100 nM siRNA. Each histogram is a representative of three independent experiments. The bar chart (D) illustrates cell cycle distribution after treatment and represents three independent experiments (n=3 ± SEM). Plk1_5 siRNA and Plk1_1 siRNA significantly increased the number of G2-arrested cells (red) in both cell lines compared to untreated cells (*$p<0.05$; ***$p<0.005$).

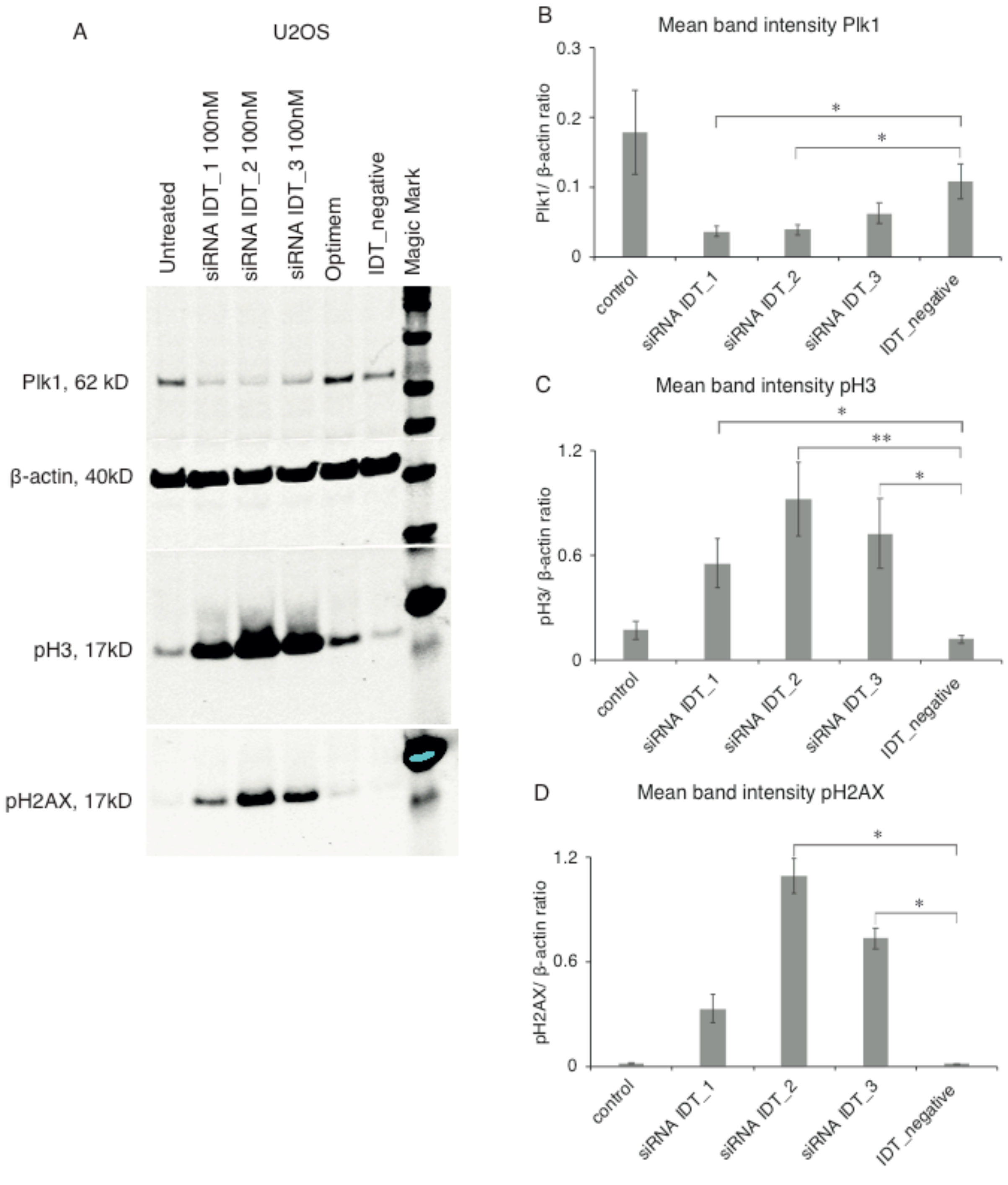

Supplementary Figure 6. Western blot analysis of siRNA sequences Plk1_IDT_1/2/3.

Full-length blots (A) displaying siRNA treatment of U2OS with three different Plk1 siRNA sequences. Magic Mark was used a ladder control. After transfer the membrane was cut into the corresponding molecular weights of indicated proteins followed by probing for the corresponding antibodies. The lower part of the membrane was stripped and probed with an antibody targeting pH2AX.The analyzed sequences induced Plk1 protein knockdown, G2 arrest

and double-strand DNA breaks compared to the negative control. The graphs B, C and D illustrate quantified protein bands of Plk1, pH3 and pH2AX on western blot membranes. Quantification was performed using Image J. Protein expression was normalized against ß-actin. Data represents mean band intensity of experiments performed as technical duplicates in three independent experiments ($n=3 \pm SEM$) ($^{*}p<0.05$; $^{**}p<0.005$).

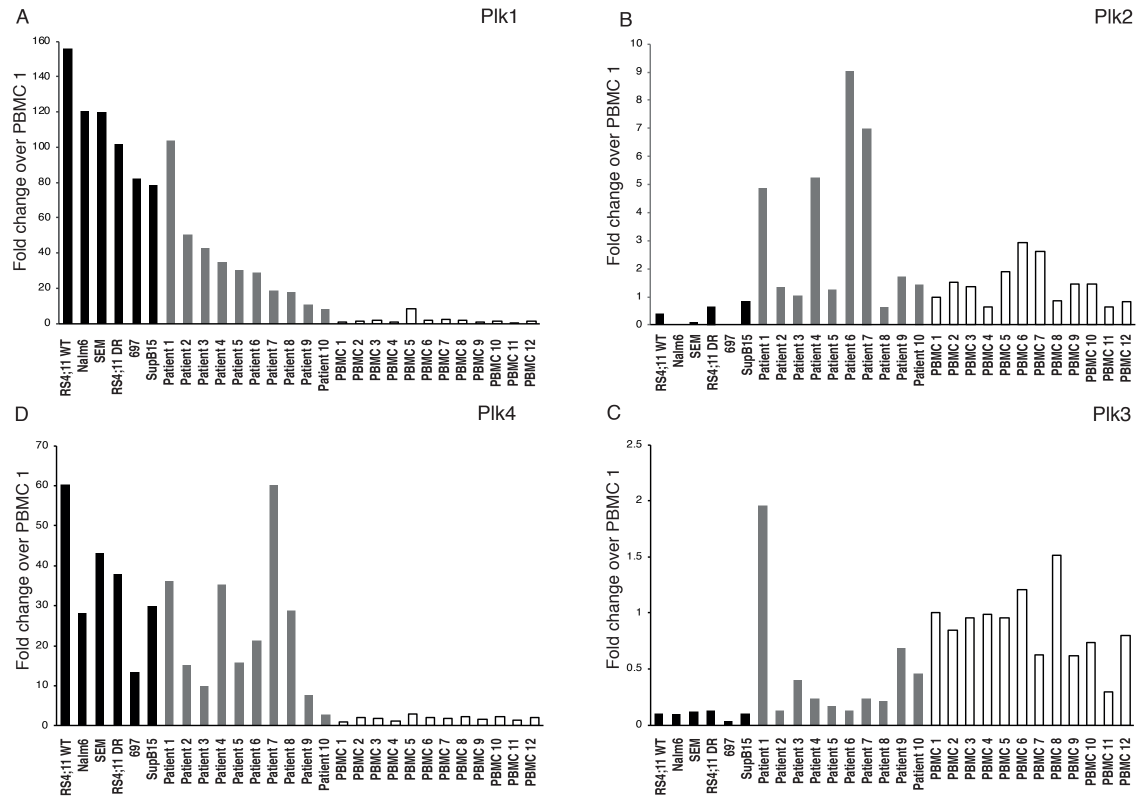

Supplementary Figure 7. Plk1-Plk4 expression in B-ALL patients relative to PBMC1.

(A) Plk1, (B) Plk2, (C) Plk3, and (D) Plk4 mRNA expression was evaluated by qRT-PCR in B-ALL cell lines (black columns), primary cells from ten pediatric B-ALL patients (grey columns) and PBMCs from twelve healthy blood donors, PBMC1 (white columns). Expression levels are relative to PBMC1. Patients were numbered according to their Plk1 mRNA expression, where Patient 1 had the highest Plk1 expression. GAPDH was used as an internal control.

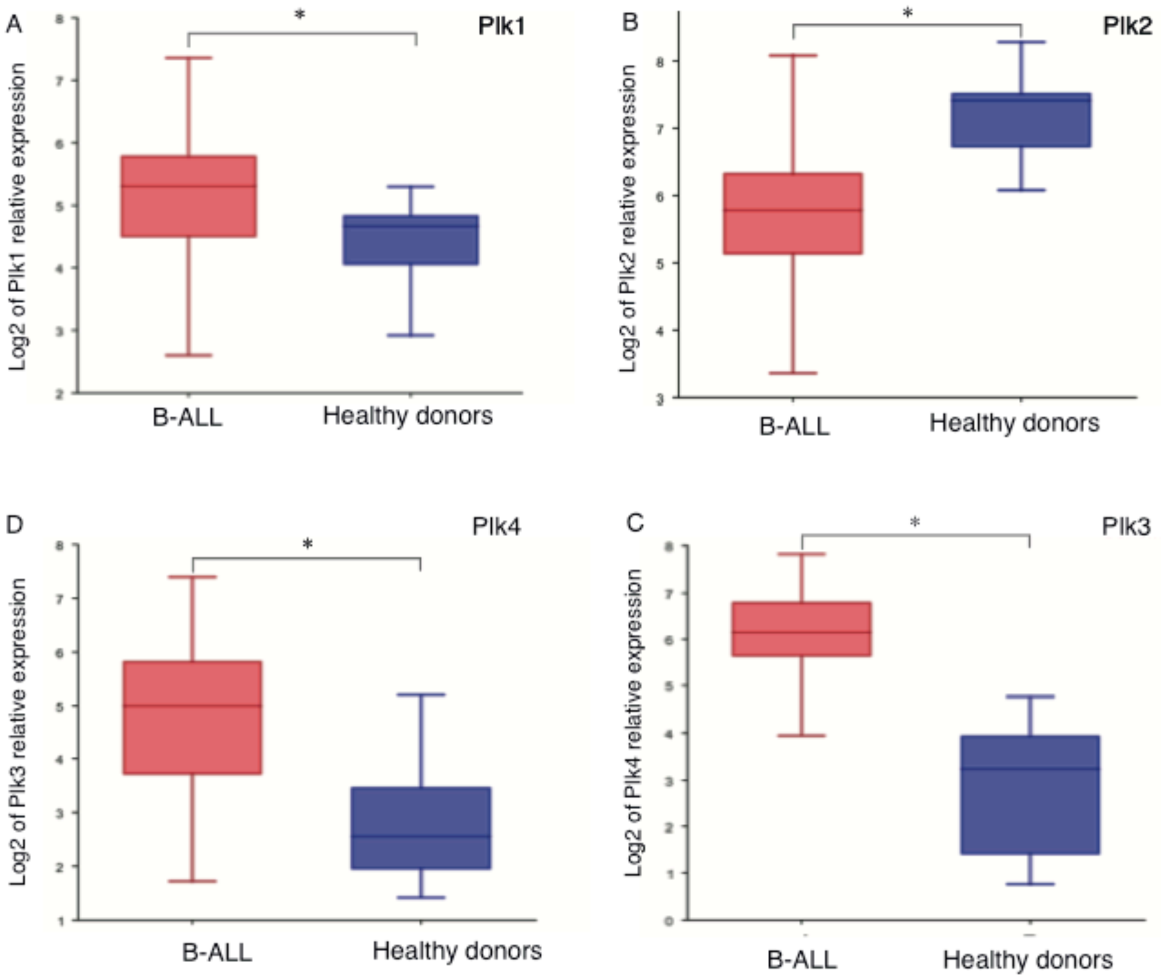

Supplementary Figure 8. Plk1-Plk4 expression in B-ALL patients compared to sorted B-cells from healthy donors.

Using R2 database, Plk1-Plk4 mRNA expression levels were analyzed in peripheral blood mononuclear cells from B-ALL patients from ALL (Murphy) dataset (red boxes, n=76) and compared to CD19+ sorted B-cells from healthy donors from the CD4- T and B cells Lauwerys dataset (blue boxes, n=9). ANOVA test showed that the expression of Plk1, Plk3 and Plk4 was significantly higher and Plk2 was significantly lower in patients with B-ALL than in normal B-cells (*$p<0.05$).

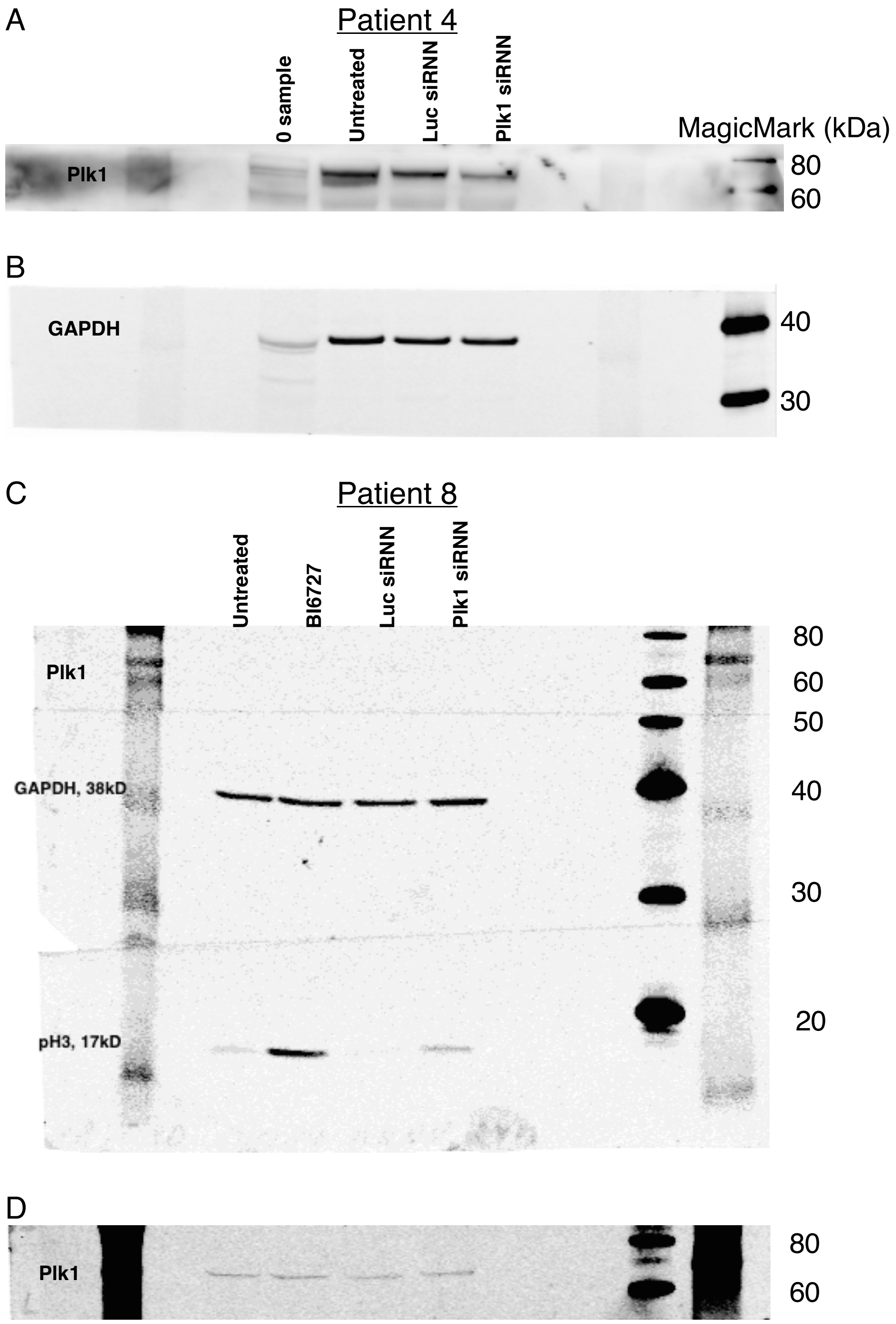

Supplementary Figure 9. Targeting Plk1 in primary cells from B-ALL patients.

Full-length blots of cropped membranes displayed in Figure 3 (A-D). SeeBlue and MagicMark were used as ladders. The blots from Figure 3A (A-B) originate from the same gel and membrane. After transfer the membrane was cut into the corresponding molecular weights of Plk1 and GAPDH followed by probing for the corresponding antibodies. However, Plk1 could not be detected by the Odyssey Infrared Imager but with Western Lightning Plus-ECL and

images were captured using Kodak M35 X-omat processor (A). The blots from Figure 3B originate from the same gel and membrane (C-D). After transfer the membrane was cut into the three parts corresponding to the molecular weights of Plk1, pH3 and GAPDH followed by probing for the corresponding antibodies (C). The Plk1 part of the membrane was reprobed with fresh antibody targeting Plk1 (D).

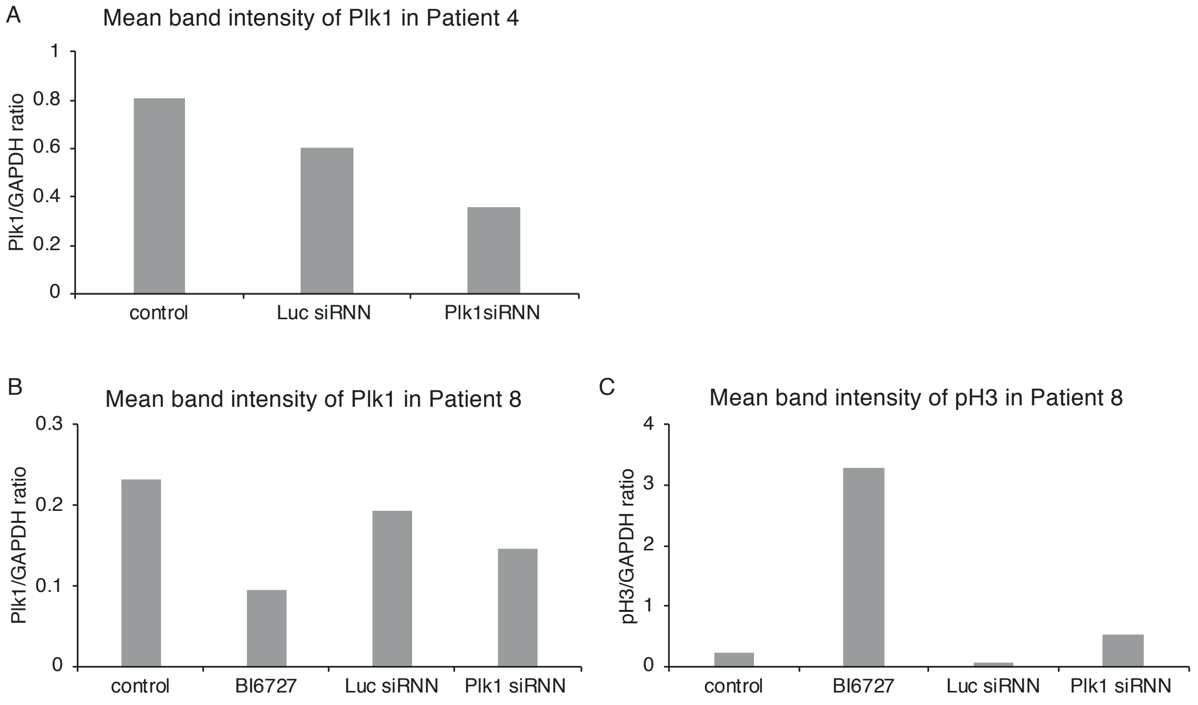

Supplementary Figure 10. Quantification of western blots performed on patient cells. The graphs illustrate quantified protein bands of western blot membranes in Patient 4 (A) and Patient 8 (B-C). Quantification was performed using Image J. Protein expression of Plk1 and pH3 was normalized against GAPDH. Data represents mean band intensity of single blots.

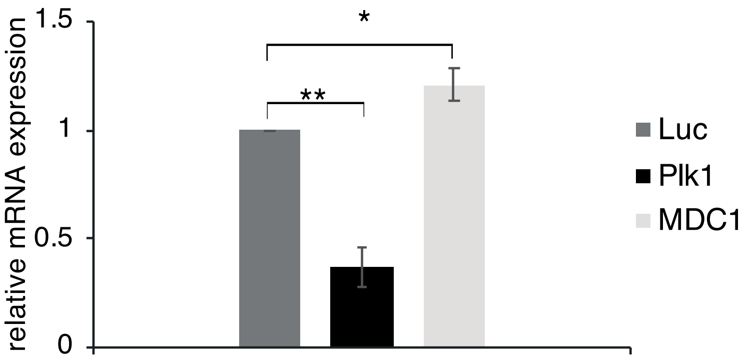

Supplementary Figure 11. Plk1 and MDC1 analysis in primary cells from pediatric B-ALL patients after siRNN treatment.

Five patient samples were treated with Luc or Plk1 siRNN at a concentration of 200 nM and analyzed by qRT-PCR 24 h after treatment. A significant increase in MDC1 mRNA expression and significant decrease in Plk1 mRNA expression was detected in Plk1 siRNN-treated samples when the patient samples were combined. GAPDH was used as internal control. Error bars represent mean ± SEM (*p<0.01; **p<0.005).
